# Applied imagination

**DOI:** 10.3389/fpsyg.2023.1275942

**Published:** 2023-11-01

**Authors:** Ed Finn, Carolina Torrejon Capurro, Michael G. Bennett, Ruth Wylie

**Affiliations:** ^1^Center for Science and the Imagination, Arizona State University, Tempe, AZ, United States; ^2^School for the Future of Innovation in Society, Arizona State University, Tempe, AZ, United States; ^3^School of Arts, Media and Engineering, Arizona State University, Tempe, AZ, United States; ^4^Institute for Experiential AI, Northeastern University, Boston, MA, United States; ^5^Mary Lou Fulton Teachers College, Arizona State University, Tempe, AZ, United States

**Keywords:** imagination, applied imagination, collaborative imagination, creativity, interdisciplinary

## Abstract

Imagination is a fundamental human capacity, and to navigate our current global challenges, we need to define and encourage the practice of imagination, or what we term “applied imagination.” In this study, we convened a series of focus groups or “virtual salons” to address three guiding questions: (1) How might we define imagination? (2) How might we (or should we) measure imagination? And (3) How might we foster imagination? Our efforts to define applied imagination highlight the crucial role imagination plays in human survival and thriving, the role of social forces in fostering or discouraging imagination, the connection between imagination and faith, and the “dark side” or maladaptive aspects of imagination. The discussions on measuring imagination were quite divided, with some salon participants arguing for the potential of indirect modes for measuring imaginative capacity while others argued that measuring imagination was functionally impossible and morally suspect. Finally, our results around fostering imagination suggest the importance of using play and humor, separating imaginative activities from the everyday, and employing constraints to prompt imaginative responses. We end with a discussion of possible directions for future research and a call to create a transdisciplinary field of imagination studies.

## 1. Introduction

Imagination: everyone knows you need it to change the world, but nobody really knows how it works. From antiquity to contemporary neuroscience, philosophers and researchers have argued that imagination plays an essential role in creating mental images and simulations that are not bounded by physical experience (Davies et al., 2011). Imagination has long been recognized as playing a special role in learning (Dewey, 1900; Greene, 2000). In the context of educational psychology, imagination is distinguished from creativity because it operates at the level of phenomenology and possibility, whereas creativity is a way of “meeting academic conventions, criteria, or constraints” (Beghetto and Schuh, 2020). But a growing contingent of researchers have come to see imagination as playing a more significant role in consciousness, as the system which supports our ongoing construction of a cognitive model of reality and ourselves within that world (Pelaprat and Cole, 2011; Asma, 2017; Pollan, 2018; Pendleton-Jullian and Brown, 2018). This paper builds on previous theoretical frameworks that distinguish imagination as a mental capacity from creativity and innovation (Liu and Noppe-Brandon, 2009; Beghetto and Schuh, 2020) to define imagination as a kind of cognitive ignition system for creativity, anticipation, and resilience. Today, we celebrate imagination in young children but often fail to foster it in formal education, and lament “failures of imagination” that lead to catastrophic errors (Weick, 2005). Imagination is not missing or inaccessible, but rather it becomes invisible, hidden behind monolithic structures of discourse and meaning-making that dominate and reinforce the status quo (Cushman, 1990; Harari, 2016). We have no trouble imagining predatory lending, toxic politics, or other destructive, yet invented and socially constructed, aspects of contemporary culture: the “crisis of imagination” we face is a crisis of autonomous, unleashed, idiosyncratic, creative imagination.

Imagination is a fundamental human capacity. Insights from evolutionary biology, psychology, neuroscience, and behavioral economics offer a framework for understanding imagination as a faculty for modeling the external world and the self that is strongly influenced by context (Byrne, 2007; Gilbert and Wilson, 2007; Seligman et al., 2016; Asma, 2017). Related work in situated cognition and extended cognition does not generally engage directly with imagination, though these concepts share common roots in educational philosophy (Stein, 1994; Pendleton-Jullian and Brown, 2018). Still, this ability remains woefully undeveloped and oftentimes ignored in traditional educational structures and professional settings. Efforts to cultivate imagination usually focus on downstream issues like field-based creativity, innovation, and skill-based workforce retraining (Liu and Noppe-Brandon, 2009). Meanwhile, climate chaos, sociopolitical upheavals, and the burgeoning human-technology frontier confront large swaths of the population with profound challenges to their identities and ways of life—our crises of imagination appear to be multiplying. As McGonigal (2022) and others have argued, we are not facing a lack of imagination, but instead a deficit in imaginative efficacy. To navigate the global challenges of the 21st century, humans will need to develop new skills for planning, problem-solving, anticipation, and adaptation. Understanding how imagination can be fostered and deployed will determine how humans can survive and flourish in the future.

The present study addresses the question of how to define and encourage the practice of imagination, or what we term “applied imagination.” This exploratory research seeks to lay groundwork for a new framework for imagination as a fundamental cognitive capacity with the following characteristics: (a) a faculty for modeling perceived reality and the self that (b) can also model other scenarios and selves and (c) is strongly influenced by context, including situated cognition and extended cognition. This project was initially planned as a scholarly conference drawing together imagination research in psychology, cognitive science, education, philosophy, and the arts and humanities. However, the COVID-19 pandemic forced us to reimagine it by engaging existing communities of practice to gather in small groups virtually and discuss shared understandings of applied imagination. We recruited nine hosts to organize virtual “salons” to convene practitioners of imagination from their own professional and creative communities. The ensuing meetings allowed intimate and sustained conversations about imagination, trading the scale of a larger gathering for a deeper set of insights into how individuals and small groups conceive of this faculty.

Our inquiry was driven by three guiding questions: (1) How might we define imagination? (2) How might we (or should we) measure imagination? (3) How might we foster imagination?

Each salon host was given broad latitude to choose whom to invite and how to structure and facilitate their sessions. The key for this project was not to regulate the process but to focus on diverse and thoughtful responses to the prompts above.

While our experimental approach to imagination is largely agnostic in terms of disciplinary perspectives, we did bring some grounding assumptions about what imagination is to bear. We assume imagination is a key element of cognition, a human potential to which everyone has access. We draw on the work of Asma, Seligman et al., and McGonigal, among others, in framing imagination as an essential tool for modeling and interpreting present reality as well as possible futures. A second important assumption is that imagination can to some extent be volitionally controlled or shaped. This underwrites our final grounding assumption: that imagination can not only be wielded consciously, but can also be exercised and strengthened like a muscle.

## 2. Methods

The host of each salon was invited to convene members of their own communities, engaging a diverse range of participants across demographic groups and cultural sectors; genders; geographies/regions/nations; occupations, disciplines, and fields of study and practice; and more (See Table 1 for a description of salon hosts and participants). These gatherings ranged from 4 to 12 participants, not counting the host, and at least one research observer.

**Table 1:** List of hosts and descriptions of the participants for each salon.

Salon host	Salon participants
Claudia Alick: Cultural producer, performer, and inclusion expert noted for her work at the intersection of gender, race, and ability-disability	Black women in the United States (gender-nonbinary guests were invited but were unable to participate because of technical difficulties)
Michael G. Bennett: Scholar of technology, law, and society	Artists, from a range of forms and media; participants also work as arts facilitators, providing support or mentoring to other artists or arts organizations
Bodhisattva Chattopadhyay: Director of the CoFUTURES project and associate professor in the Department of Culture Studies and Oriental Languages at the University of Oslo	An interdisciplinary, globally diverse group of researchers and practitioners connected with the CoFUTURES research group; participants represent disciplines ranging from literary studies, dance, and film to architecture, political science, and journalism
Fabrice Guerrier: Science fiction and fantasy author and founder of the production company Syllble	A diverse group of activists, policy experts, social entrepreneurs, and artists/creators, all living and/or working in Washington, DC
Corey S. Pressman: Anthropologist, artist, educator, and faculty member in the School of Nursing at the University of Portland	Writers and artists with expertise in bioelectronics, Renaissance and Baroque Italian art, poetry, and neuropsychology
Lisa Kay Solomon: Designer in residence at Stanford University’s d.school, with a focus on methods for futures and design thinking	Educators and facilitators working in a range of settings on issues of innovation, creativity, and agency
Laura Tohe: Emeritus professor of English at Arizona State University	Community leaders, artists, and scholars representing four Indigenous nations: Diné/Navajo, O’otham/Gila River, Nde, and Seminole Muskogee
Troy L. Wiggins: Science fiction and fantasy author, publisher of *FIYAH Magazine of Black Speculative Fiction*, educator, and community organizer	Black artists, music producers, activists, facilitators, public servants, and educators in the Memphis, Tennessee community
Ytasha Womack: Afrofuturist author and theorist, dancer and choreographer, and filmmaker	A diverse group of artists, creatives, culture workers, and marketers, many of whom are multi-hyphenate creators with elements of their practice in music or writing

We organized a facilitator workshop with our hosts to review the goals of the project, provide examples of strategies for structuring sessions and presenting activities, and solicit their feedback. Each host had agency to plan and offer invitations for their salon individually, based on this shared strategic understanding of the project goals. We encouraged hosts to align the structure, form, and timing of their salons to the needs and preferences of their participants and communities. Our rationale for having each host lead the framing and invitations for their session was to broaden participation, inviting people and perspectives beyond the usual circles and communities engaged by the Center for Science and the Imagination (CSI). This framing builds on a body of work at CSI on the theme of collaborative imagination (Finn and Wylie, 2021).

The salons were hosted in April, May, and June 2021 as virtual meetings through Zoom. One member of our research team attended each of the salons. The roles that our team members assumed, and the intensity of their participation in the salons, varied depending on context. At the beginning of each salon, the researcher present provided a brief project overview and discussed participant privacy and consent. In some cases the researcher then served as a silent observer throughout. In others, they participated actively in the ensuing conversation when invited by the host or when they shared expertise or identity with participants. In a few cases, the researcher left the session completely after the introductory remarks.

The salons were recorded, and audio, video, and text-chat transcripts were shared with the research team. After the salon, each host submitted a reflection document where they described their event: the group they convened and the communities represented by participants, the structure of the event, and brief summaries of the participants’ perspectives on defining, measuring, and cultivating imagination.

With help from our graduate student researcher, our preliminary data analysis included qualitative analysis of salon video recordings, hosts’ reflections, and real-time observation of participants’ interactions. We coded the data based on our three central themes: (a) defining imagination, (b) measuring imagination, and (c) fostering imagination. During the first round of coding, we observed each salon recording and noted when participants made points that aligned with one or more of our themes. We then grouped these observations in a first attempt to identify common themes across the nine salons. In the next step, we coded information from the post-salon reflection documents each host completed. Next, we identified common themes between our researcher notes and the hosts’ reflections (see Section 3). Finally, building on this analysis, all members of the research team convened to identify themes and patterns that resonated with—and/or meaningfully departed from—previous CSI work around our three core concepts: defining, measuring, and cultivating applied imagination. Because the data reflected perspectives from multiple viewpoints, after coding and compiling the results, we invited the salon hosts to review our findings and provide feedback or offer alternative interpretations.

## 3. Results

### 3.1. Defining imagination

The definitions of imagination generated in the salons reflect the polysemy and multivalence often attributed to the term. Discussions about defining imagination in our salons revealed that participants had strong feelings and commitments about certain aspects and manifestations of imagination, and that they found it to be an essential, if somewhat quicksilver, element of their work as artists, researchers, organizers, leaders, and more, playing an important role in their conceptions of the self.

#### 3.1.1. Imagination as fundamental capacity

Participants across salons defined imagination as something essential, a foundational aspect of cognition to which all humans have access. Several participants contended that imagination is key to survival—a component of resilience that enables us to escape the tribulations of our lived realities, and to develop ideas, competencies, and strategies to resist or change oppressive or challenging conditions.

Some participants metaphorized imagination as a “muscle,” suggesting that imaginative capacity can be honed, providing individuals and groups with greater access to their innate imaginative resources. Others referred to imagination as a “gift,” a skill or talent that is perhaps unique to humans, but that requires exercise in order to use effectively and rewardingly. Applying terminology from cognitive science, another salon group discussed imagination as the “default network,” referring to the medial frontoparietal network, which is theorized to be active when an individual is not focused on a specific task and refers to states such as “wakeful rest” or “daydreaming” that are ideationally generative but not necessarily task- or goal-directed.

Consonant with the definition of imagination as a fundamental capacity, several salon groups accounted for people’s different experiences with imagination, and perceived “failures” or “lacks” of imagination, in terms of social and cultural forces discouraging use of, or engagement with, imagination. Many participants reflected that just as imagination can be exercised and encouraged, it can be blocked or discouraged in restrictive schooling or work settings, in families and communities, and by exhausting or oppressive societal conditions. Participants pointed to how systemic social forces such as cultural bias, racism, classism, and colonization can hinder people’s access to their imaginative resources, thereby encouraging concrete, status-quo-focused modes of thought and action that reinforce hierarchies of domination and subordination. These are examples of imagination being strongly influenced by context.

#### 3.1.2. Imagination and creativity

The conflation of imagination and creativity is a leitmotif in cognitive, psychological, and learning-science research on these terms and their educational, organizational, and societal applications. Some participants contended that our keyword for these salons, “applied imagination,” was synonymous with creativity, while others positioned imagination as a precursor to creativity—imagination as an activity that generates ideas, and creativity as a subsequent activity of bringing those ideas into being in the world. One participant described the relationship vividly as a way of transforming one energy into another: creativity takes the cathexis of imagination and transmutes it into a digital or material composition such as a poem or a piece of software.

Imagination was associated more often with the ideational, the immaterial, the subconscious and prelingual—perhaps with the numinous—whereas creativity was associated with the concrete, the logistical, problem-solving, artisanal craft, and excellence in realization. As such, there might be said to be a Cartesian quality to the way that people make sense of the wavy, fluid distinction between these terms, in that imagination is a mental capacity while creativity is typically enacted through some physical medium.

#### 3.1.3. Connections to spirituality and alternative states

Following on the numinous character of the imagination in contradistinction to creativity, several salon participants defined imagination in relation to the irreal, spirituality, and altered states of consciousness (modelling other scenarios and other selves). Referring to a common definition of imagination from vernacular and research literature, participants described imagination as the act of seeing, feeling, and sensing something that is not literally or materially present. This could refer to the mundane as well as the novel—that is, to memory and projection to familiar circumstances, as well as to the speculative and the impossible.

Several participants described imagination as deeply connected to faith and spirituality, as well as to folklore, which was positioned as particularly well-attuned to expressing imaginary states and styles of connection, potent imagery, and free association. One salon host speculated that these connections may provide a compelling lens to understand organized religion as one of the most durable and potent forms of collective imagination in human history. Others connected the imagination to dreams and dream states, especially in the types of unexpected, intuitive connections and juxtapositions that are possible when in an imaginative “flow” and in a dream.

#### 3.1.4. The dark side of imagination

While most accounts of imagination, in our workshops and in the broader cultural discourse, stress its positive attributes, participants across three of our salons emphasized that imagination is not innately prosocial or constructive. One salon group discussed how imaginative achievements have undergirded systems of collective brutality, providing rationales and cultural frames for prisons and other penal institutions. Another focused on how particular imaginary frames can be imposed by dominant groups upon oppressed groups, leading to distortions that perpetuate colonization and subordination. Yet another considered how the imagination’s connection to altered states of consciousness could be maladaptive and unhealthy, and how people living with mental illness might have negative experiences that are simultaneously imaginatively rich and ornate.

### 3.2. Resistance to measurement

While our participants were enthusiastic about sharing and building upon one another’s definitions of imagination, they met the question of measuring imagination with skepticism and resistance. Some believed that measuring imagination, or imaginative capacity, is functionally impossible, and others believed that the act of measurement was morally suspect, and could serve to deform or curtail people’s access to their imaginative resources. For many participants imagination is mercurial, elusive, associated with the irreal and subconscious. As such, the act of measuring imagination might be perceived as a fundamental misunderstanding of its nature, or an act of investigative violence to a cognitive feature that is understood as standing in opposition to positivistic regimes of assessment and validation.

This resistance helps to clarify the emergent definition of imagination arising from these salons. Participants perceived efforts to measure imagination as a form of bureaucracy, and positioned imagination as inimical to hierarchy, repetitive process, and institutionalization (a challenging finding for institutions, including our own, invested in attempting to foster and support imaginative capacity). For some participants, this bureaucratization serves to limit imagination—that is, the act of calcifying imagination for purposes of assessment might interfere with the imaginative processes, skills, and characteristics being measured. In other words, these efforts might fundamentally alter the context of situated cognition and extended cognition in which imagination occurs.

In two salons, participants explicitly connected efforts to measure imagination with dynamics of colonization, where measurement would concretize and channel energy and resources toward a limited definition of imagination set by and serving the interests of the dominant group. They stressed that the study of imagination ought to be decolonized, and that our efforts to both define and measure imagination need to be mindful of valuing a diversity of cultural contexts and knowledge systems, especially those emanating from Indigenous groups and communities in the Global South. One salon group explicitly described efforts to measure imagination as supportive of dominant white culture—which is particularly poignant since many participants defined imagination as a resource for survival and resilience in dehumanizing and deeply unequal social orders. Participants across most of our salons were more receptive to assessing imagination indirectly. Modes for indirect measurement included assessing creative or practical outputs (e.g., problem-solving tasks or artwork), or measuring iteration, diversity of perspectives, and number of “adjacent possible” solutions or permutations explored by either individuals or groups working collaboratively. Others suggested measuring impact by observing how interventions or texts shape thinking and behavior in individuals and communities.

Among our participants, people who work as educators were most receptive to measuring imagination, because having assessments available is a key element of effective teaching and cultivating imaginative practices. This underscores the importance of context in these measurements. Measuring imagination carries many risks, but it also has the potential to make this fundamental human capacity more visible to structures of power. Questions of which incentive structures motivate measurement, how people participate in those regimes, and how such measurements might be used all impact the potential efficacy and ethics of any attempt to measure imagination.

Some salon participants emphasized that imagination is not a concrete and fixed characteristic, and that its manifestations, and variations across cultures and contexts, are fluid, irreducibly diverse, and emergent. Imagination should be conceived of and evaluated as a process, and one that is socially situated and often interactive. As such, efforts to measure and assess imagination might respond to resistance by focusing on evaluating the effectiveness of tools, methods, and processes for unlocking imaginative capacity, rather than to quantify and measure, for example, the amount or intensity of imagination.

### 3.3. Cultivating imagination

Salon participants expressed great enthusiasm for a variety of strategies to cultivate imagination and foster imaginative capacity among individuals and groups.

One strategy, expressed across several salons, is the need for separation from the mundane, or day-to-day routines and demands, in order for people to tap into their imaginative capacity. This “room of one’s own” insight stresses the need to make time and space for imagination. Participants identified a variety of methods to achieve this separation from the often creativity-numbing rigidity of everyday life, including walking in nature, physical exercise, and meditation.

A related theme is the role of play and humor in cultivating imagination—again, drawing on an insight from our discussion of defining imagination around the connection between imagination and the irreal and the irrational. This also dovetails with skepticism about bureaucratic and hierarchical settings as militating against people’s free and full access to their imaginative resources, and emphasizes that space ought to be made for divergent thinking, fun, and lines of conversation, thought, and recreation that are not directly tied to specific problems or instrumental goals.

Building on the role of humor and play, several salon groups discussed how imagination can be used to reject dominant paradigms such as capitalism and colonialism. One group likened this rejection of the dominant via imagination to the idea of “Black joy,” by which author Tracey M. Lewis-Giggetts (2022) connects experiences of joy and pleasure to resilience and resistance to systemic racism. Two groups suggested that we might liberate our notions of imagination by decoupling them from the concept of “innovation” and its connotations of opening new avenues for generating wealth. Another group considered how decolonization may be a form of imagination that works to reveal truths to create change. In these figurations, imagination is more potent when it eschews instrumentalization.

Participants in several salons expressed the importance of constraints in enabling people to effectively access and use their imaginations. These constraints were positioned as helping to direct imaginative energies and provide a basis upon which individuals might iterate and develop ideas. Artists, especially those who create under self-imposed limits, such as jazz musicians or poets working within the bounds of specific forms such as haiku, are exemplary. The experimental musician Bjork could be speaking for many artists when she says, “the less room you give me/the more space I’ve got” (Bjork, 1997). But research in education and pedagogy (Tromp and Baer, 2022) and business management (Acar et al., 2019) also lends support to the idea that something less than pure freedom can enrich the imagination. There is a delicate tension to constraints, since they can channel focus and build resilience, but also serve as barriers or needlessly sap energy, depending on context.

### 3.4. Imagination and mindfulness

One final theme that emerged in several of the salons was the relationship or overlap between mindfulness, futures thinking, and imagination. As a critical element in foresight or anticipation, imagination is a fundamental capacity for thinking ahead or envisioning possible future scenarios, and in this way, imagination is a precursor to futures thinking. The role of imagination in relation to mindfulness is more complex: several participants talked about the importance of holding space or clearing away distractions to allow imagination to work, including practices like meditation or engaging in other tasks to “take their mind off” a problem. This raises the interesting question of how individuals imagine or narrate their own sense of consciousness, and the power of imaginative storytelling in creating or enacting the conditions for mindfulness.

## 4. Discussion

While our participants shared a broad range of reactions to the theme of imagination, we have identified four key findings in the data. 1) Imagination is both important and elusive. Participation in these salons spanned many different disciplines and industries, but the conversations consistently identified imagination as very important to many people in their work, yet also hard to define. This supports our initial framing of imagination as a fundamental cognitive capacity that is widely used but not always widely recognized. 2) To address this challenge, participants repeatedly defined imagination through its absence or in opposition: imagination against hegemony, in resistance to the status quo, or as a protean counterforce to more visible barriers. These contextual factors play an important role in making imagination visible. 3) The metaphor of force and counterforce, or a mechanics of imagination, guides us to another way in which imagination was repeatedly described: as a kind of “work” or set of practices that can be honed and improved upon, even if that work is primarily mental or cognitive. 4) Discussions of imaginative practice repeatedly led to variations on the idea of imaginative capacity: the agency of individuals or groups of people to construct and share their own imaginative perspectives and ideas. Imagination is therefore an important vehicle for change precisely because it allows individuals to conceive of altered scenarios and altered selves.

## 5. Conclusion

Imagination is an amorphous subject to address, particularly if the intention is not to instrumentalize this versatile cognitive capacity. The authors acknowledge the influence of our own positionalities and prior experiences on how we have reported these findings and how they resonate with our own understandings of imagination, both theoretically and as a practical tool for creativity, resilience, and futures thinking. The format of these salons also sets clear limitations to the evidence gathered here—future research might explore different configurations of participants, considering varying conditions such as gender, race, ethnicity, professional practice, and prior familiarity with other participants, and formalizing structured questions or activities consistently across all groups. One key takeaway from our findings is how difficult the cognitive capacity of imagination is to disentangle from other capacities for action and reflection, or indeed from consciousness itself.

Nevertheless, the energy and conviction of these conversations is undeniable. Practitioners, scholars, and leaders of many different stripes all agree that imagination is essential to their work, and largely concur that imagination can be practiced and enhanced, both individually and collectively.

These results suggest several important avenues for further research. How do we advance the contentious question of measuring imagination? Indirect measurements of impact and efficacy are one possibility, and could build on existing measures of creativity, futures consciousness, and resilience. But any such approaches must also contend with how to hold space for diverse practices and cultures of imagination while still working to make this core human capacity more visible. The relationships among imagination, spirituality, states of altered consciousness, and organized religion are all fascinating and relatively unexplored areas for future study.

Looking forward, there is so much yet to learn about how the imagination works at an individual and collective level. There is exciting research going on in cognitive science, in psychology, in philosophy and the creative arts. But very few people are trying to connect these dots or study imagination as a shared experience. The challenge we see is to continue building the field of imagination studies, drawing on these disciplines and the manifold ways that people cultivate and deploy imagination every day around the world.

## Data availability statement

The original contributions presented in the study are included in the article/supplementary material, further inquiries can be directed to the corresponding author.

## Ethics statement

The studies involving humans were approved by Arizona State University’s Institutional Review Board. The studies were conducted in accordance with the local legislation and institutional requirements. The participants provided their written informed consent to participate in this study. Written informed consent was obtained from the individual(s) for the publication of any potentially identifiable images or data included in this article.

## Author contributions

EF: Writing – original draft. RW: Writing – original draft. MGB: Writing – review & editing. CTC participated in data collection, coding, analysis, and early drafts of the procedure and results.
